# Development and Validation of a Simplified Five-Variable Score for Predicting Massive Transfusion in Blunt-Trauma-Predominant Patients

**DOI:** 10.3390/jcm15166344

**Published:** 2026-08-17

**Authors:** Soon Ki Min, Byungchul Yu, Giljae Lee, Kangkook Choi, Youngmin Kim, Wu Seong Kang, Mina Lee

**Affiliations:** Department of Trauma Surgery, Gachon University Gil Medical Center, Incheon 21556, Republic of Korea; minsk0611@gilhospital.com (S.K.M.); kane2123@gilhospital.com (B.Y.); nonajugi@gilhospital.com (G.L.); choikangkook@gilhospital.com (K.C.); skyofmay@gilhospital.com (Y.K.); wuseongkang@gilhospital.com (W.S.K.)

**Keywords:** massive transfusion, trauma, prediction score, hemorrhagic shock, ionized calcium, ABC score, TASH score, TBSS

## Abstract

**Background/Objectives:** Early identification of trauma patients requiring massive transfusion (MT) is essential for timely activation of massive transfusion protocols. Existing models have limitations: the Assessment of Blood Consumption (ABC) score includes penetrating injury, which limits its use where blunt trauma predominates; the Trauma-Associated Severe Hemorrhage (TASH) score requires numerous weighted variables; and the Traumatic Bleeding Severity Score (TBSS) requires blood pressure measured after crystalloid infusion. This study aimed to develop and validate a simplified MT prediction score using only variables available during initial assessment. **Methods:** We retrospectively analyzed 1462 adult trauma patients (366 MT, 1096 non-MT) treated at a regional trauma center in Korea between January 2020 and December 2025. Candidate predictors were identified by logistic regression, and five candidate models were compared. The final model, designated the Simplified Massive Transfusion (SMT) score, was compared with the ABC, TASH, and Modified TBSS scores in the same complete-case cohort using the area under the receiver operating characteristic curve (AUC) and the paired DeLong test. **Results:** The SMT score comprises five variables—systolic blood pressure ≤ 110 mmHg, lactate ≥ 4.0 mmol/L, ionized calcium ≤ 1.05 mmol/L, positive FAST, and pelvic fracture—each assigned one point (range, 0–5). In a common cohort of 1352 patients, the SMT score (AUC 0.846, 95% CI 0.822–0.870) performed similarly to the TASH score (0.841; *p* = 0.668) and significantly better than the Modified TBSS (0.821; *p* = 0.015) and the ABC score (0.783; *p* < 0.001). MT probability increased with increasing score, from 2.8% at a score of 0 to 82.9% at a score of 4. The observed rate at a score of 5 was 81.2%, but only 32 patients had this score. **Conclusions:** The SMT score, based on five readily available variables, showed discriminatory ability comparable to the TASH score and superior to the Modified TBSS and ABC scores. Since none of its components depends on the mechanism of injury, it may be broadly applicable across trauma populations. Further external validation in multicenter cohorts, especially those with a greater prevalence of penetrating trauma, is needed.

## 1. Introduction

Hemorrhage remains one of the leading causes of preventable death after trauma [1]. Because most hemorrhagic deaths occur within the first few hours after injury [1,2], early identification of patients likely to bleed significantly is central to damage control resuscitation. Massive transfusion protocols (MTPs) allow rapid, balanced delivery of blood products and early correction of trauma-induced coagulopathy. Timely MTP activation improves outcomes, whereas delayed activation increases mortality [3,4,5].

The difficulty lies in predicting, during the initial assessment, which patients will actually require massive transfusion (MT). Delayed activation may result in preventable hemorrhagic death; unnecessary activation wastes limited blood products and increases cost [6]. Several prediction models have been proposed to address this problem, including the Assessment of Blood Consumption (ABC) score [7], the Trauma-Associated Severe Hemorrhage (TASH) score [8], and the Traumatic Bleeding Severity Score (TBSS) [9].

Each of these models has limitations. The ABC score requires only four variables and is simple to calculate, but one of its components is penetrating injury, which limits its usefulness in systems where blunt trauma predominates. In a validation cohort that directly examined this issue, patients with an ABC score of 2 or higher had a penetrating mechanism in 59% of cases, compared with only 20% among those with a lower score [10]. Because a positive score is achieved more easily by penetrating than by blunt injury, the ABC score may systematically underperform in populations where blunt trauma predominates.

The TASH score performs well but requires numerous physiological, laboratory, and injury-related variables, making it cumbersome to apply during initial assessment [8]. The TBSS achieves high accuracy but requires systolic blood pressure measured after rapid infusion of 1000 mL of crystalloid [9]. Determination is therefore delayed until fluid resuscitation is complete, which conflicts with the purpose of early protocol activation. A modified version using blood pressure on arrival was later proposed for this reason [11].

Model performance depends on the distribution of injury mechanism in the population where it is applied. Blunt trauma predominates in many trauma systems, and in the Republic of Korea a nationwide analysis of the Korean Trauma Data Bank reported that more than 95% of registered trauma cases resulted from blunt mechanisms [12]. Data from our own institution reflect this pattern: among 32,025 patients treated at our regional trauma center between 2014 and 2023, penetrating injury accounted for only 6.9% of cases [13]. In such settings, a score that assigns a point for penetrating injury forfeits that component in most patients. This is not a limitation of any one country but of applying a mechanism-dependent score to a population in which that mechanism is uncommon.

We therefore aimed to develop and validate a simplified prediction score for MT, using only variables available during the initial trauma assessment. We hypothesized that a small number of carefully selected variables could achieve discrimination comparable to more complex models while remaining practical for early bedside use. We deliberately excluded mechanism of injury from the final score. A score built only from physiological, laboratory, and anatomical measures carries no assumption about how the injury occurred, and can therefore be tested in any trauma population.

## 2. Materials and Methods

### 2.1. Study Design and Patient Selection

This retrospective study was conducted at the Regional Trauma Center of Gachon University Gil Medical Center, Incheon, Republic of Korea. Adult trauma patients (≥18 years) who presented to the emergency department between January 2020 and December 2025 were included.

**Figure 1:**
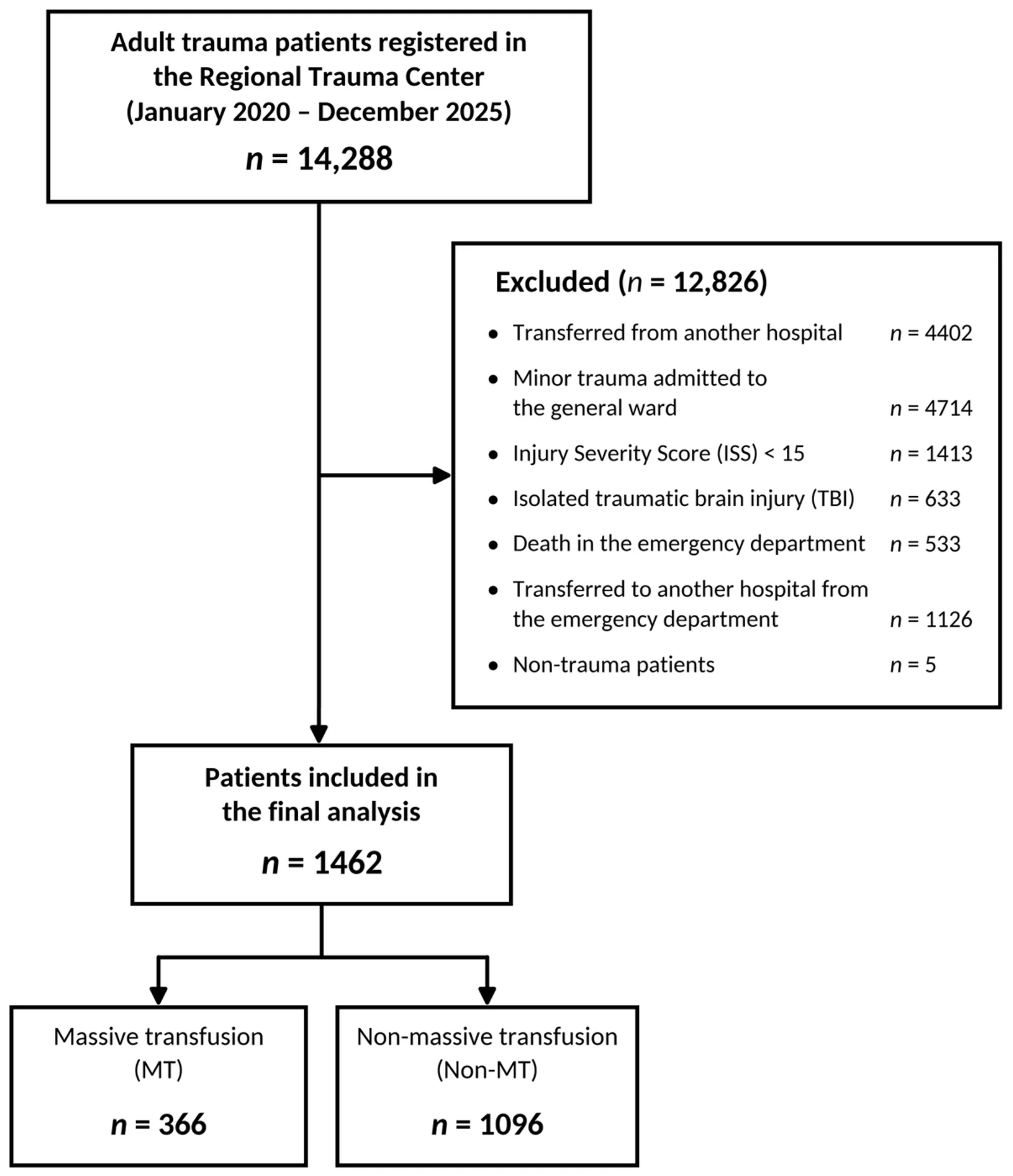
Patient flow diagram. Adult trauma patients registered at the Regional Trauma Center between January 2020 and December 2025 were screened for eligibility. After exclusion criteria were applied, 1462 patients were included in the final analysis: 366 in the massive transfusion (MT) group and 1096 in the non-MT group.

Patients were excluded if they were transferred from another hospital, admitted to the general ward for minor trauma, had an Injury Severity Score (ISS) < 15, had isolated traumatic brain injury, died in the emergency department before initial evaluation was complete, were transferred to another hospital from the emergency department, or had a non-traumatic diagnosis (Figure 1).

No patient in this cohort arrived with a pre-existing do-not-resuscitate order or a documented limitation of care at presentation.

The study protocol was approved by the Institutional Review Board of Gachon University Gil Medical Center (IRB No. GDIRB2026-234). Informed consent was waived because of the retrospective design.

### 2.2. Data Collection

Clinical data were extracted from the Korean Trauma Data Bank (KTDB) and electronic medical records by trained investigators.

Variables were grouped into demographics (age, sex), injury characteristics (mechanism, ISS, Abbreviated Injury Scale [AIS] score, pelvic fracture, femur fracture), physiological variables (systolic and diastolic blood pressure, heart rate, respiratory rate, oxygen saturation, body temperature, Glasgow Coma Scale [GCS] score), laboratory findings (pH, base excess, lactate, ionized calcium, hemoglobin, platelet count), imaging findings (FAST), and clinical outcomes (hospital stay, ICU stay, in-hospital mortality). Pelvic fractures were classified as AO/OTA (tile) type A, B, or C from the AIS code assigned by trauma surgeons during registry coding. Fractures classified as type B or C were regarded as unstable, and type A as stable.

Arterial blood gas analysis was performed on a GEM Premier 3500 analyzer (Instrumentation Laboratory, Bedford, MA, USA) located within the trauma resuscitation room. Lactate and ionized calcium were measured in arterial blood from the same sample, obtained during the initial assessment on arrival, and both results were available within two minutes. Neither variable required a separate venipuncture, an additional laboratory order, or transport to the central laboratory. Hemoglobin and platelet count were obtained from a complete blood count on a separate venous sample. No fixed time window was specified for sampling, although the sample was drawn during the initial evaluation in all patients.

All patients were direct admissions, since interhospital transfers were excluded. Administration of blood products is outside the scope of practice of emergency medical services in the Republic of Korea, and no patient received any blood product before hospital arrival. Prehospital fluid volumes are not recorded in our registry.

Patients with missing systolic blood pressure were excluded before model development. Systolic blood pressure was considered essential to the prediction model.

### 2.3. Institutional Massive Transfusion Protocol

Our institution operates a standardized MTP. Activation follows the attending trauma surgeon’s overall judgment, informed by systolic blood pressure below 90 mmHg, heart rate above 120/min, positive FAST, penetrating wound, pelvic fracture, or contrast extravasation on imaging. These criteria serve as guidance rather than as fixed triggers. Anesthesiology staff assume the activating role intraoperatively. Blood products are delivered in a balanced ratio, targeting red cells to fresh frozen plasma close to 1:1. Adjuvant measures include tranexamic acid, calcium chloride, and active warming. Complete blood count, coagulation profile, fibrinogen, and arterial blood gas analysis are repeated during resuscitation. Transfusion is tapered once hemoglobin exceeds 10 g/dL, prothrombin time falls below 18 s, activated partial thromboplastin time below 35 s, and platelet count exceeds 150 × 10^3^/μL. Whole blood was not used at any point during the study period; all patients received component therapy. Calcium chloride is administered after protocol activation, whereas ionized calcium in this study was measured on arrival, before any calcium supplementation.

### 2.4. Outcome Measure

Patients were divided into an MT group and a non-MT group. Massive transfusion (MT) was defined as transfusion of ≥4 units of packed red blood cells (pRBC) within 4 h of emergency department arrival, or ≥10 units within 24 h [14,15].

A patient meeting either criterion was classified as MT. Of the 366 MT patients, 358 met the 4-h criterion, 149 met the 24-h criterion, and 141 met both. All transfusions were counted from emergency department arrival, and units administered in the operating room or angiography suite within the relevant window were included.

### 2.5. Development of the Prediction Model

Candidate predictors were first identified by univariable logistic regression. Variables with significant association were entered into a multivariable model. Multicollinearity was assessed using the variance inflation factor (VIF). Variables with substantial collinearity were excluded before multivariable modeling.

Continuous variables were dichotomized using cutoff values from receiver operating characteristic (ROC) curve analysis with the Youden index. For clinical use, these cutoffs were rounded to practical values—for example, systolic blood pressure ≤ 110 mmHg instead of the derived ≤109.5 mmHg, and ionized calcium ≤ 1.05 mmol/L instead of ≤1.02 mmol/L.

Five candidate models were developed by progressively removing variables with limited incremental value. Variables were prioritized by immediate availability and clinical relevance, not statistical significance alone. Discrimination and simplicity were both considered when selecting the final model. The final model was designated the Simplified Massive Transfusion (SMT) score. Mechanism of injury was excluded from the final score by design. A score composed only of physiological, laboratory, and anatomical measures carries no assumption about how the injury occurred and may therefore be suitable for evaluation in trauma populations with different distributions of injury mechanism.

### 2.6. Statistical Analysis

Continuous variables are presented as median (IQR) and compared using the Wilcoxon rank-sum test. Categorical variables are presented as number (%) and compared using the chi-square test or Fisher’s exact test, as appropriate.

Sample size varied across analyses because different variables had different amounts of missing data. Baseline characteristics and univariable analysis used all available data for each variable. Multivariable analysis and candidate model development used complete-case data for the variables required in each model.

The ABC score was calculated from its four components—penetrating injury, systolic blood pressure ≤ 90 mmHg, heart rate ≥ 120/min, and positive FAST—and considered positive at a score ≥ 2. The TASH score was calculated as originally described and considered positive at ≥8.

The original TBSS requires systolic blood pressure measured after rapid infusion of 1000 mL of crystalloid, a value that is not recorded in routine practice and cannot be recovered retrospectively. We therefore calculated the Modified TBSS, which applies the same scoring algorithm using systolic blood pressure on arrival [11], and considered it positive at ≥14. The Modified TBSS scores the number of positive FAST regions among the six regions specified in the original score: pericardium, right and left thorax, perihepatic space, perisplenic space, and pelvis. Our registry records FAST findings in four categories, which do not map directly onto these six regions. Two investigators therefore reviewed the original ultrasound reports and medical records of every FAST-positive patient, and the number of positive regions was recounted according to the original definition. Patients recorded as FAST-negative in the registry were assigned zero positive regions.

To compare the SMT score with the TASH, Modified TBSS, and ABC scores, all four were evaluated in the same complete-case cohort, restricted to patients with data available for every variable required by any of the four scores. Discrimination was assessed by the area under the ROC curve (AUC). AUCs were compared using the paired DeLong test. Internal validation was performed by bootstrapping with 1000 replicates. Optimism-corrected estimates of discrimination and the calibration slope were obtained by Harrell’s method. Because the cutoffs for continuous variables were derived in the same cohort, a further bootstrap was performed in which the Youden-based cutoffs were re-derived within each replicate. Calibration was assessed by plotting observed against predicted probability at each score level, with a Hosmer-Lemeshow-type test across levels.

Sensitivity analyses were performed using two alternative definitions of MT: ≥10 units within 24 h alone, and ≥4 units within 4 h alone.

All analyses were performed in R (version 4.3.1; R Foundation for Statistical Computing, Vienna, Austria). Two-sided *p* < 0.05 was considered statistically significant.

### 2.7. Use of Generative AI

During the preparation of this manuscript, the authors used ChatGPT (OpenAI, San Francisco, CA, USA; GPT-5) and Claude (Anthropic, San Francisco, CA, USA; Claude Opus 5) to improve the readability and language of the text. These tools were also used to check statistical code for errors. The study design, the analyses, and the interpretation of the results were performed by the authors. All code was executed by the authors in R (version 4.3.1), and every numerical result reported in this manuscript was verified by the authors against the source data. Generative AI was not used to generate, alter, or impute any patient data. After using these tools, the authors reviewed and edited the content as needed and take full responsibility for the content of this publication.

## 3. Results

### 3.1. Patient Characteristics

During the study period, 14,288 adult trauma patients were registered. After exclusion criteria were applied, 1462 patients were included in the final analysis: 366 in the MT group and 1096 in the non-MT group (Figure 1).

Baseline characteristics are shown in Table 1. The median interval from injury to emergency department arrival was 60 min (IQR 38–60) and did not differ between groups (*p* = 0.364); 79.5% of patients arrived within 1 h and 95.2% within 2 h. Among transfused patients, the median interval from arrival to the first transfusion was 17 min (IQR 10–42) in the MT group and 126 min (IQR 64–284) in the non-MT group (*p* < 0.001).

Compared with the non-MT group, the MT group had lower systolic and diastolic blood pressure, higher heart rate and respiratory rate, and lower GCS scores (all *p* < 0.001). Laboratory findings also differed: the MT group had lower pH, hemoglobin, platelet count, and ionized calcium, and higher lactate and base deficit (all *p* < 0.001).

The MT group had a higher Injury Severity Score (29 vs. 22, *p* < 0.001). Severe injury (AIS ≥ 3) was most frequent in the thorax (929 patients, 63.5%), followed by the head and neck (777, 53.1%), extremity and pelvis (540, 36.9%), and abdomen (475, 32.5%). Compared with the non-MT group, the MT group had more frequent severe injury of the abdomen (55.7% vs. 24.7%), extremity and pelvis (54.4% vs. 31.1%), and thorax (68.0% vs. 62.0%).

A positive FAST was more common in the MT group (37.7% vs. 11.0%, *p* < 0.001), as was each individual region: hemothorax (6.3% vs. 1.0%), pneumothorax (16.4% vs. 5.8%), abdomen (21.3% vs. 4.5%), and pelvis (3.6% vs. 0.7%) (all *p* < 0.001). Pelvic fracture (48.4% vs. 23.3%) and femur fracture (27.3% vs. 15.4%) were also more common (both *p* < 0.001). Among 432 patients with pelvic fracture, 211 were classified as AO/OTA type A, 185 as type B, and 36 as type C.

Missing data are summarized in Supplementary Table S1. Lactate was missing in 28 patients (1.9%) and ionized calcium in 31 (2.1%). Neither variable was missing in any patient who received massive transfusion, and all missing values occurred in the non-MT group (both *p* < 0.001). Systolic blood pressure showed the opposite pattern, and was missing in 21 of 1096 non-MT patients (1.9%) and 49 of 366 MT patients (13.4%; *p* < 0.001).

### 3.2. Predictors of Massive Transfusion

In univariable analysis, nearly all examined variables were associated with MT, except for age (*p* = 0.261) (Table 2).

Variables with independent clinical relevance were entered into the multivariable model. Diastolic blood pressure, oxygen saturation, body temperature, individual FAST subregions, pelvic fracture type, femur fracture, pH, base excess, and platelet count were excluded before multivariable modeling because of collinearity with variables already included.

After adjustment, penetrating injury, systolic blood pressure, heart rate, respiratory rate, GCS, lactate, hemoglobin, ionized calcium, positive FAST, and pelvic fracture remained independent predictors of MT (Table 3). Age and male sex were not significant in the multivariable model.

Hemoglobin remained a significant independent predictor (adjusted OR 0.79, *p* < 0.001) but was not included in the final score. Ionized calcium was selected instead, as it captures coagulation- and hemodynamics-related information not reflected by hemoglobin alone.

### 3.3. Development of the Final Prediction Model

ROC analysis with the Youden index was used to determine optimal cutoff values for continuous predictors (Table 4). At the level of individual variables, systolic blood pressure (AUC 0.792), lactate (AUC 0.781), and ionized calcium (AUC 0.787) showed relatively strong discrimination, whereas heart rate (AUC 0.693) and GCS (AUC 0.621) performed less well. These three variables were retained in the final model.

Using these cutoff values, rounded to clinically conventional numbers, five candidate models were developed and compared (Table 5; Supplementary Table S2). Model A, which included all nine candidate variables, had the highest discrimination (AUC 0.864). Model B, adding penetrating injury to a five-variable set of Model C, achieved a similar AUC (0.850). Model C, the five-item model without penetrating injury, showed only a marginal reduction (AUC 0.845). Because penetrating injury is uncommon in our blunt-trauma-predominant population, it was excluded from the final score to preserve generalizability. Models D and E, with four and three variables, had lower AUCs of 0.838 and 0.802.

Considering both discrimination and simplicity, Model C was selected as the final model, hereafter referred to as the Simplified Massive Transfusion (SMT) score. Internal validation by bootstrapping with 1000 replicates showed minimal optimism. For a logistic model using the five binary items, the apparent AUC was 0.853 and the optimism-corrected AUC 0.851. For the unweighted integer score applied in practice, the apparent and optimism-corrected AUCs were both 0.845. When the Youden-based cutoffs were re-derived within each bootstrap replicate, the apparent AUC was 0.856 and the optimism-corrected AUC 0.852. The calibration slope was 0.986 and the Brier score 0.119 (Supplementary Table S3).

### 3.4. Comparison with Existing Prediction Models

The SMT score, TASH, Modified TBSS score and ABC score were compared in the same complete-case cohort (*n* = 1352), restricted to patients with data available for all variables required by the four scores (Table 6).

**Figure 2:**
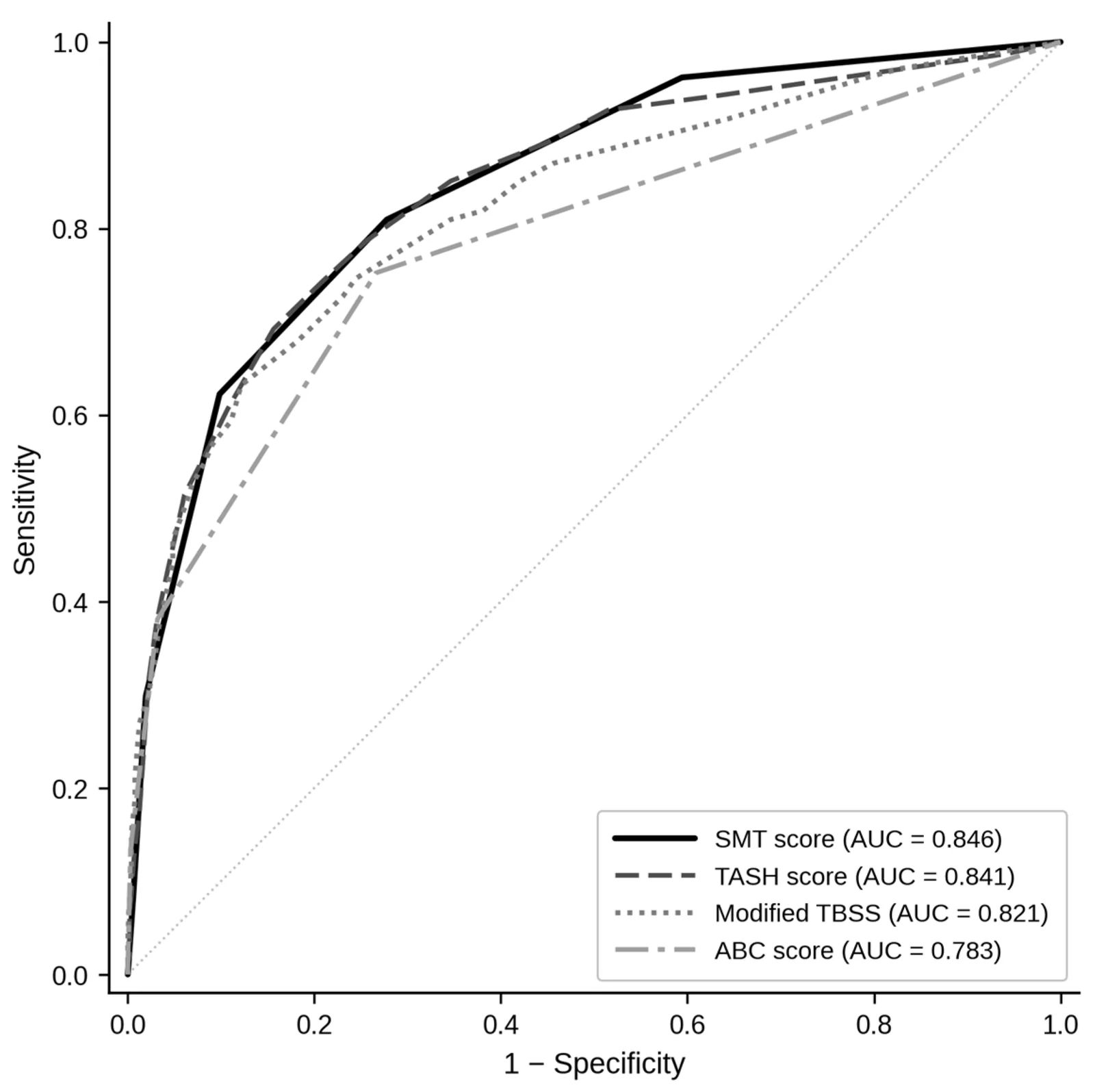
Receiver operating characteristic curves comparing the Simplified Massive Transfusion (SMT) score, the Trauma-Associated Severe Hemorrhage (TASH) score, the Modified Traumatic Bleeding Severity Score (TBSS), and the Assessment of Blood Consumption (ABC) score for predicting massive transfusion, evaluated in the same complete-case cohort (*n* = 1352). The diagonal line indicates no discrimination (AUC = 0.50). AUC, area under the curve.

The SMT score had an AUC of 0.846 (95% CI, 0.822–0.870), the TASH score 0.841 (0.815–0.868), the Modified TBSS 0.821 (0.793–0.850), and the ABC score 0.783 (0.754–0.812) (Figure 2).

By paired DeLong test, the SMT score did not differ from the TASH score (*p* = 0.668) but significantly outperformed the Modified TBSS (*p* = 0.015) and the ABC score (*p* < 0.001). The TASH score also outperformed the ABC score (*p* < 0.001), and the Modified TBSS outperformed the ABC score (*p* = 0.010). The difference between the TASH score and the Modified TBSS did not reach significance (*p* = 0.058).

Results were unchanged under alternative definitions of MT (Supplementary Table S4). Using ≥10 units within 24 h alone (120 events), the AUCs were 0.830 for the SMT score, 0.821 for TASH, 0.792 for the Modified TBSS, and 0.789 for ABC. Using ≥4 units within 4 h alone (307 events), they were 0.849, 0.846, 0.826, and 0.784. Discrimination was slightly lower under the more restrictive definition, but the ordering of the scores was preserved.

### 3.5. Clinical Performance of the SMT Score

**Figure 3:**
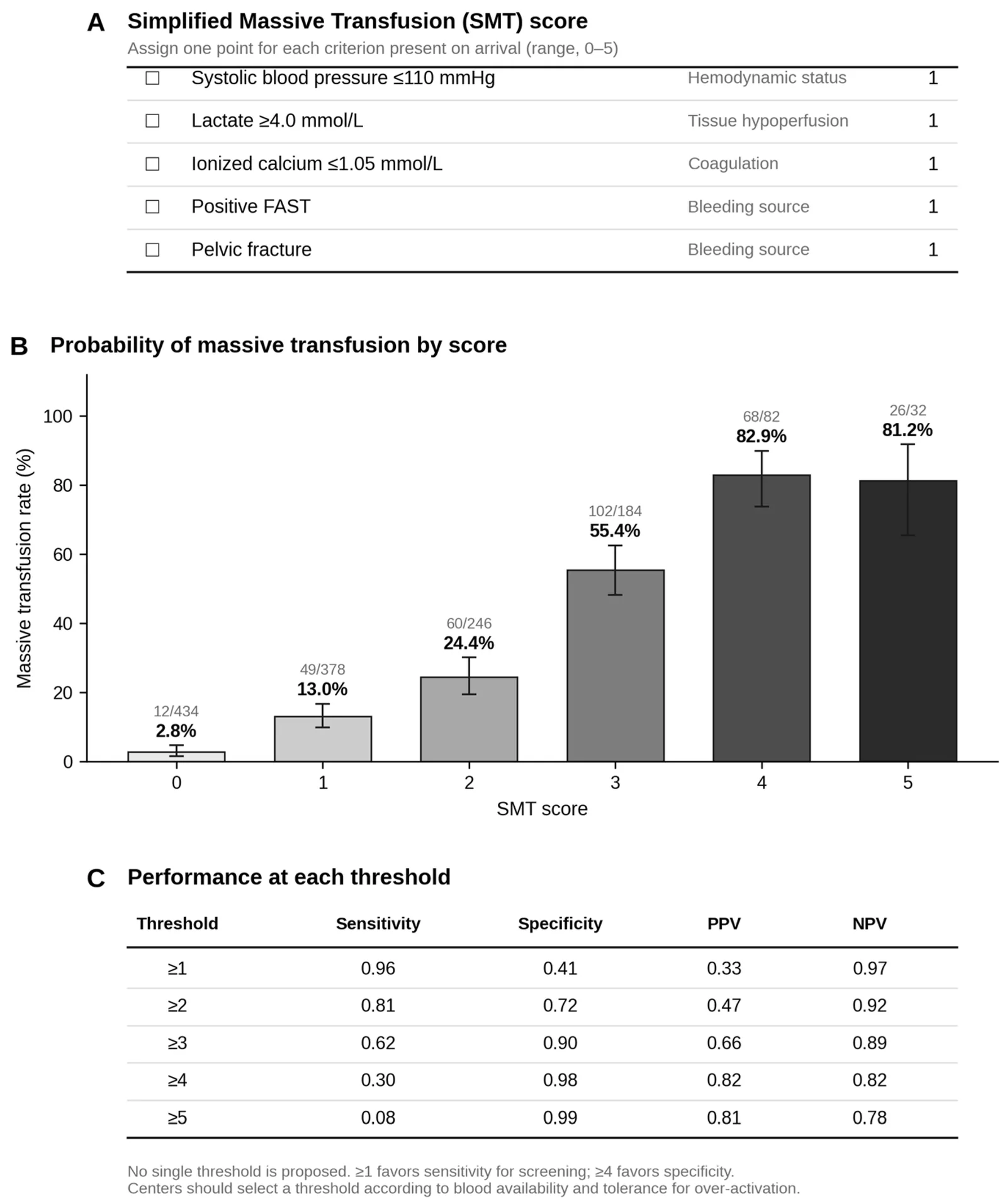
The Simplified Massive Transfusion (SMT) score as a bedside tool. (**A**) Score criteria, each contributing one point, with the pathophysiological domain represented. (**B**) Massive transfusion rate at each score (*n* = 1356); values above each bar indicate the proportion of patients who received massive transfusion (events/total), and error bars represent 95% confidence intervals. (**C**) Sensitivity, specificity, positive predictive value, and negative predictive value at each threshold.

**Figure 4:**
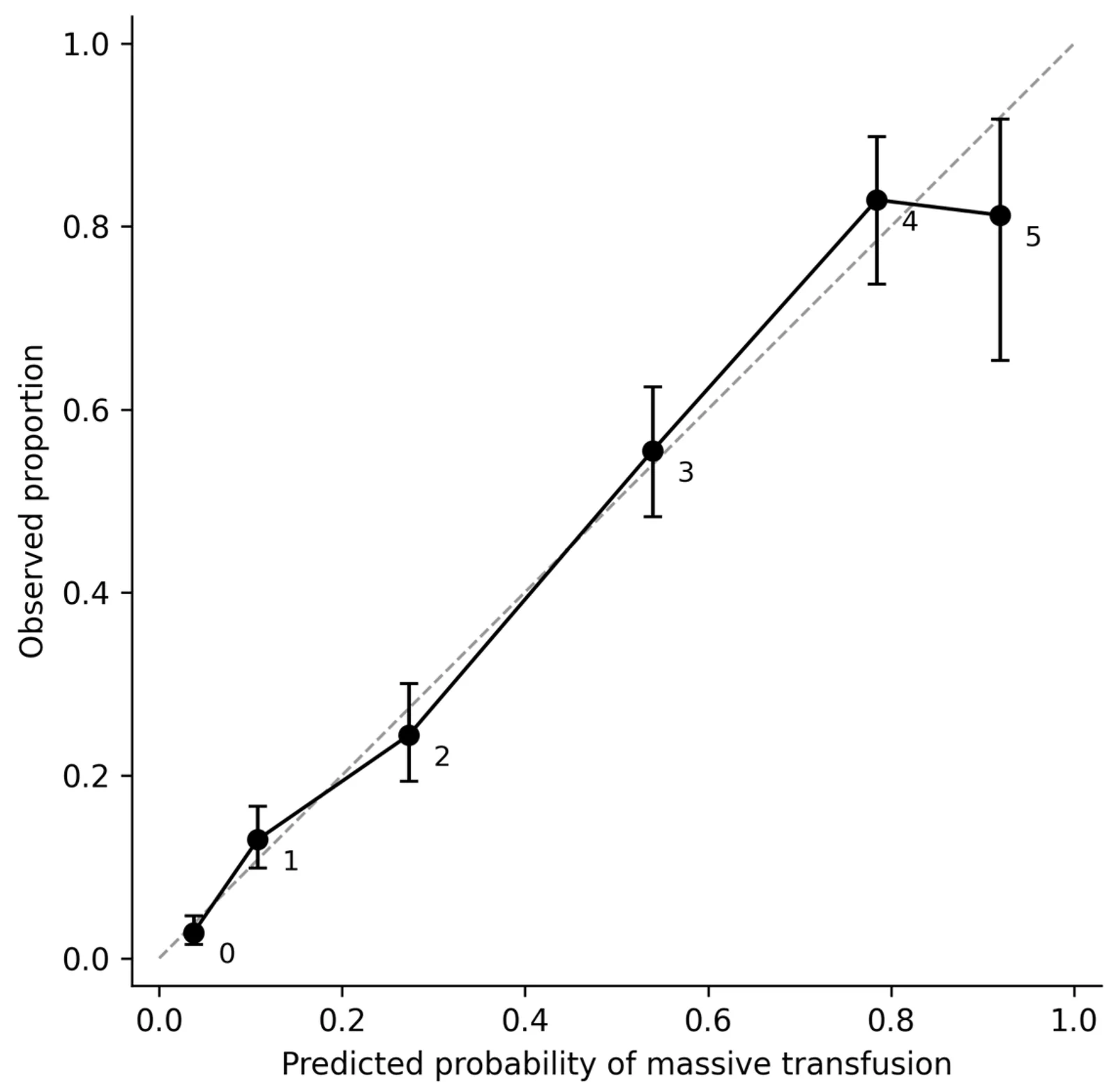
Calibration of the Simplified Massive Transfusion (SMT) score. Observed proportion of patients receiving massive transfusion is plotted against the predicted probability at each score level, with 95% confidence intervals. Numbers beside each point indicate the score. The dashed line represents perfect calibration.

The scoring system assigns one point to each of the five variables (total range, 0–5). Figure 3 presents the criteria, the corresponding probability of MT, and the operating characteristics at each threshold in a single bedside format. MT probability generally increased with increasing score (Figure 3B). Among patients with a score of 0, only 12 of 434 (2.8%) received MT. This proportion rose sharply with increasing score: 13.0% (49/378) at a score of 1, 24.4% (60/246) at 2, 55.4% (102/184) at 3, 82.9% (68/82) at 4, and 81.2% (26/32) at 5. The SMT score can therefore be used to stratify risk across several levels, and not only as a binary threshold. Calibration was close across the lower and middle of the range (Figure 4). Observed and predicted probabilities were 2.8% and 3.7% at a score of 0, 13.0% and 10.8% at 1, 24.4% and 27.3% at 2, 55.4% and 53.9% at 3, and 82.9% and 78.5% at 4. At a score of 5 the observed proportion was 81.2% against a predicted 91.9%, although only 32 patients had this score and the confidence interval was wide (65.4–91.8%). A Hosmer-Lemeshow-type test across score levels gave *p* = 0.039, driven by this uppermost category.

Sensitivity, specificity, positive predictive value, negative predictive value, and accuracy at each cutoff (≥1 through ≥5) are shown in Table 7. A low cutoff (≥1) gave high sensitivity (96.2%) with low specificity (40.6%), which may be suited to screening for patients who could require MT. A higher cutoff (≥4 or above) gave specificity exceeding 98%, which may help identify patients with a high probability of requiring MT. We did not propose a single cutoff. Performance at each threshold is presented so that clinicians can select a cutoff according to their own setting, depending on whether sensitivity or specificity is more important.

## 4. Discussion

We developed and validated a simplified five-variable score, the SMT score, to predict massive transfusion (MT) in trauma patients. The final model uses five variables available during initial assessment: systolic blood pressure, lactate, ionized calcium, FAST findings, and pelvic fracture. In the same complete-case cohort, the SMT score showed discrimination comparable to the TASH score, and higher than that of the Modified TBSS and the ABC score (Table 6). The observed MT rate generally increased with increasing SMT score, although the rate at a score of 5 was slightly lower than that at a score of 4 because of the small number of patients in the highest-score category.

Our aim was not to maximize statistical performance by including as many variables as possible, but to build a tool that could be applied quickly during initial trauma evaluation. We therefore compared models with progressively fewer variables. The most complex model (Model A) had the highest AUC, but the improvement over the five-item model was small—0.864 versus 0.845. Model B, which added penetrating injury to a five-variable set, showed a similarly small gain (AUC 0.850). Because penetrating injury is uncommon in our setting, we considered that this added complexity did not yield a corresponding improvement in performance.

Early recognition of patients likely to need MT is one of the central challenges in trauma care. Timely MTP activation improves hemorrhage control and survival, whereas delayed recognition postpones balanced transfusion and definitive hemorrhage control. A useful prediction model must therefore be both accurate and fast to apply.

Although the SMT score uses only five variables, its discrimination was similar to that of the TASH score, which requires more variables and weighted scoring. This result suggests that a score can be simplified without a large loss of predictive performance if the variables are selected appropriately.

### 4.1. Pathophysiological Rationale

Variables were selected on the basis of clinical relevance as well as statistical significance. We aimed to represent distinct pathophysiological domains of hemorrhage, rather than several variables reflecting the same underlying process.

Systolic blood pressure is one of the most established indicators of hemorrhagic shock and is included in both the ABC and TASH scores [7,8]. Hypotension develops relatively late, after compensatory mechanisms fail, but its onset reliably signals clinically significant hemorrhage.

FAST and pelvic fracture provide complementary anatomical information. Among the recognized sources of major hemorrhage after injury—external bleeding, the extremities, thorax, abdomen, and the pelvis/retroperitoneum—FAST reliably identifies only intraperitoneal free fluid [16]. Bleeding confined to the retroperitoneal space, from a pelvic fracture, can therefore remain undetected even when FAST is negative; prior series have reported concomitant retroperitoneal hemorrhage in a substantial proportion of patients with blunt abdominal trauma and pelvic injury, with ultrasound performing poorly in this region [17]. Together, FAST and pelvic fracture therefore cover two of the most common and otherwise easily missed bleeding sources in blunt trauma.

Among laboratory variables, we selected lactate and ionized calcium because they reflect different physiological processes. Lactate indicates tissue hypoperfusion and often rises before hypotension becomes evident, making it useful during compensated shock. Base excess showed a similar association in univariable analysis but largely reflects the same process as lactate, so we chose lactate as the more direct and widely available point-of-care measure.

Hemoglobin was also a significant independent predictor (Table 3) but was not included in the final score, because concentration changes lag behind acute blood loss until fluid shifts produce hemodilution.

Ionized calcium provides different information. In patients with major hemorrhage, ionized calcium falls through several mechanisms, including chelation by citrate contained in transfused blood products, metabolic acidosis from hypoperfusion, and intracellular calcium shift following tissue injury [18]. Because calcium is a cofactor at several steps of the coagulation cascade, its depletion accompanies the onset and progression of trauma-induced coagulopathy; a prospective cohort of 591 critically ill trauma patients found that admission ionized calcium independently predicted the need for multiple transfusions [19], and a separate observational analysis reported an association between hypocalcemia and the severity of traumatic coagulopathy [20].

Whether hypocalcemia contributes causally to early mortality after injury has recently been questioned. In a multicenter cohort of 2141 transfused trauma patients, hypocalcemia was no longer associated with 24-h mortality after adjustment for acidosis, coagulopathy, and hypothermia (adjusted OR 1.11, 95% CI 0.84–1.48), and admission ionized calcium may therefore reflect injury severity and hypoperfusion rather than acting as an independent driver of outcome [21]. In this cohort, ionized calcium showed the second highest individual discrimination among continuous predictors (AUC 0.787), remained independent after adjustment (adjusted OR 0.37 per 0.1 mmol/L), and is reported within two minutes on the same arterial sample as lactate. We therefore regard it as an early marker of the coagulation and circulatory derangement accompanying major hemorrhage, and our results do not address whether calcium supplementation improves outcome.

Together, the five variables span four domains: hemodynamic status (systolic blood pressure), anatomical bleeding source (FAST, pelvic fracture), tissue hypoperfusion (lactate), and coagulation (ionized calcium). This combination may account for the performance of the score despite its small number of variables.

### 4.2. Clinical Implications

Compared with existing models, the SMT score combines the strengths of the ABC and TASH scores while avoiding their main limitations. The ABC score requires only four variables but includes penetrating injury as one component, which limits its use in blunt-trauma-predominant systems; prior validation studies have shown that a positive ABC score is achieved far more easily in penetrating than in blunt injury [10]. The TASH score performs well but requires numerous weighted variables, which limits its practicality during initial evaluation. In its original form, the TASH score combines seven variables with unequal weights and a maximum of 28 points: hemoglobin contributes up to 8 points, systolic blood pressure up to 4, base excess up to 4, heart rate up to 2, positive FAST up to 3, complex pelvic or long-bone fracture up to 6, and male sex 1 point [8]. Calculating this score at the bedside requires simultaneously recalling multiple weighting schemes rather than a simple count, which is less practical during the first minutes of resuscitation than a score in which each variable contributes a single point. The TBSS takes a different approach, achieving high accuracy with five variables but requiring systolic blood pressure after infusion of 1000 mL of crystalloid [9]. Determination is therefore delayed until fluid resuscitation is complete. The Modified TBSS was proposed to address this [11], and in our cohort it performed less well than the SMT score despite using the same number of variables. This finding suggests that the selection of variables is more important than their number or weighting.

All five components are available during the initial trauma assessment and early resuscitation, without additional calculators or delayed laboratory results, allowing the score to be calculated within minutes of arrival. Both laboratory components derive from a single arterial blood gas sample analyzed within the resuscitation room, with results available in two minutes. This distinguishes the SMT score from the TASH score, which requires hemoglobin and base excess, the former obtained from a separate complete blood count. No component of the SMT score requires a test that would not otherwise be performed during the initial evaluation of a severely injured patient. Early identification of high-risk patients may support earlier MTP activation, earlier blood bank communication, and earlier preparation for hemorrhage control; delays in these steps have been associated with worse outcomes in prior studies [3,4].

Our institutional MTP was originally based on the ABC score for its simplicity, but pelvic fracture and contrast extravasation were added because penetrating injury is uncommon in our population. The same limitation that prompted this local modification motivated the present study, and pelvic fracture was retained as a component of the final score.

The SMT score is intended to support, not replace, clinical judgment. Physician experience and institutional protocol remain central to transfusion decisions. Nonetheless, an objective, simple bedside tool may improve consistency, particularly for less experienced clinicians or in high-volume trauma centers.

Our center used component therapy throughout the study period, and whole blood was not used at any point. Two recent phase 3 trials found no advantage of prehospital whole blood over component therapy [22,23]. Our findings therefore apply to a component-therapy setting, and the comparison of prediction scores was not influenced by differences in transfusion strategy.

### 4.3. Strengths

This study has several strengths. First, all five variables in the final score are available during initial trauma assessment, without additional imaging or complex calculation. Second, the model was developed through stepwise simplification, comparing models with progressively fewer variables rather than selecting variables by statistical significance alone. Third, the final score was compared directly with the ABC, TASH, and Modified TBSS scores in the same complete-case cohort, allowing a fair head-to-head comparison. Fourth, internal validation by bootstrapping showed negligible optimism, including when the cutoffs were re-derived within each replicate. Finally, the cohort was drawn from six consecutive years of a regional trauma center where blunt trauma predominates, reflecting current practice in this setting.

### 4.4. Limitations

Several limitations should be noted.

This was a retrospective, single-center study. External validation in multicenter cohorts is needed before wider use.

The score was developed in a population in which 96.9% of injuries were blunt. None of the five components depends on the mechanism of injury, and the score may therefore be applicable to populations with a higher proportion of penetrating injury. However, this was not evaluated in the present study and requires further validation.

A total of 533 patients died in the emergency department before the initial assessment was complete, and 70 further patients had missing systolic blood pressure. Their exclusion may have introduced survivorship bias. Blood pressure was missing in 13.4% of MT patients compared with 1.9% of non-MT patients, which suggests that the excluded patients were more severely injured. The findings should therefore be interpreted as applying to patients who survived long enough to undergo a complete initial evaluation, and may not represent those who died shortly after arrival.

Lactate and ionized calcium were sampled during the initial evaluation, but a fixed time window was not specified. Prehospital crystalloid infusion may lower ionized calcium by dilution. Transport time was short in our system and no patient received prehospital blood products, but the volume of fluid administered is not recorded in our registry. In addition, the score requires a blood gas analyzer that reports both lactate and ionized calcium, which is not available in all centers.

Injury time in the trauma registry is estimated rather than measured, and the recorded values were concentrated at 30 and 60 min. The prehospital interval reported in this study should therefore be regarded as an approximate value.

The MTP activation criteria at our institution overlap with the components of the prediction scores, and an influence on transfusion decisions cannot be excluded. The overlap is largest for the ABC score, because its components form the basis of the institutional criteria. This may have favored the ABC score in the present comparison.

Calibration was acceptable at the lower and middle score levels, but was less reliable at scores of 4 or higher, in which the number of patients was small.

Sensitivity and specificity at the cutoffs presented should be interpreted with caution. The threshold for the SMT score was derived from the same cohort, whereas the thresholds for the ABC, TASH, and Modified TBSS scores were those originally reported. This does not affect the comparison of AUC, which is independent of the threshold.

Finally, this study did not establish a single threshold for MTP activation. The choice of cutoff may depend on institutional practice and should be evaluated in future prospective studies.

## 5. Conclusions

We developed and validated a simplified five-item score, the SMT score, for predicting massive transfusion in adult trauma patients. Despite using fewer variables than the TASH score, it showed comparable discrimination, and it outperformed both the Modified TBSS and the ABC score in direct comparison. The score combines hemodynamic, anatomical, metabolic, and coagulation-related variables into a single bedside tool, and all five components are obtained during the primary survey. The score was developed in a blunt-trauma-predominant cohort, but no component depends on mechanism of injury. Prospective multicenter studies, including in populations with a higher proportion of penetrating trauma, are needed to validate the score externally and to assess its effect on MTP activation and outcomes.

## Figures and Tables

**Table 1:** Baseline characteristics according to massive transfusion.

Characteristic	Overall (*n* = 1462)	Non-MT (*n* = 1096)	MT (*n* = 366)	*p*-Value
**Demographics**				
Age, years	55 (38, 65)	55 (39, 66)	54 (36, 64)	0.246
Sex, male	1104 (75.5%)	843 (76.9%)	261 (71.3%)	0.037
**Mechanism of injury**				
Blunt	1417 (96.9%)	1081 (98.6%)	336 (91.8%)	<0.001
Penetrating	45 (3.1%)	15 (1.4%)	30 (8.2%)	
**Prehospital and early care**				
Injury-to-ED interval, min	60 (38, 60)	60 (38, 60)	60 (38, 60)	0.364
ED arrival to first transfusion, min ^a^	55 (14, 148)	126 (64, 284)	17 (10, 42)	<0.001
**Vital signs**				
Systolic blood pressure, mmHg	131 (106, 156)	139 (118, 162)	96 (75, 123)	<0.001
Diastolic blood pressure, mmHg	83 (67, 100)	87 (74, 102)	60 (48, 81)	<0.001
Heart rate, beats/min	93 (78, 112)	90 (77, 105)	110 (90, 129)	<0.001
Respiratory rate, breaths/min	20 (20, 25)	20 (19, 24)	24 (20, 29)	<0.001
Oxygen saturation, %	97 (94, 98)	97 (95, 98)	95 (89, 98)	<0.001
Body temperature, °C	36.3 (36.0, 36.7)	36.4 (36.0, 36.8)	36.0 (35.5, 36.6)	<0.001
Glasgow Coma Scale	14 (9, 15)	15 (10, 15)	13 (6, 15)	<0.001
**Laboratory**				
pH	7.39 (7.32, 7.43)	7.40 (7.35, 7.44)	7.32 (7.20, 7.40)	<0.001
Base excess, mmol/L	−1.5 (−5.2, 0.9)	−0.5 (−3.0, 1.5)	−5.9 (−11.2, −2.5)	<0.001
Lactate, mmol/L	2.8 (1.9, 4.8)	2.5 (1.6, 3.7)	5.3 (3.1, 8.2)	<0.001
Ionized calcium, mmol/L	1.08 (1.03, 1.12)	1.09 (1.06, 1.13)	1.01 (0.93, 1.07)	<0.001
Hemoglobin, g/dL ^b^	13.4 (12.0, 14.5)	13.7 (12.5, 14.7)	12.2 (10.5, 13.6)	<0.001
Platelet count, ×10^3^/μL	239 (195, 288)	243 (198, 291)	229 (187, 275)	<0.001
**Injury severity**				
Injury Severity Score	24 (19, 33)	22 (19, 29)	29 (22, 38)	<0.001
**AIS ≥ 3 by body region**				
Head and neck	777 (53.1%)	629 (57.4%)	148 (40.4%)	<0.001
Face	38 (2.6%)	23 (2.1%)	15 (4.1%)	0.058
Thorax	929 (63.5%)	680 (62.0%)	249 (68.0%)	0.046
Abdomen	475 (32.5%)	271 (24.7%)	204 (55.7%)	<0.001
Extremity and pelvis	540 (36.9%)	341 (31.1%)	199 (54.4%)	<0.001
**FAST and fracture findings**				
**Positive FAST findings**				
Hemothorax	34 (2.3%)	11 (1.0%)	23 (6.3%)	<0.001
Pneumothorax	124 (8.5%)	64 (5.8%)	60 (16.4%)	<0.001
Abdomen	127 (8.7%)	49 (4.5%)	78 (21.3%)	<0.001
Pelvis	21 (1.4%)	8 (0.7%)	13 (3.6%)	<0.001
Any positive FAST	259 (17.7%)	121 (11.0%)	138 (37.7%)	<0.001
Pelvic fracture, AO/OTA type	432 (29.5%)	255 (23.3%)	177 (48.4%)	<0.001
Type A (intact) ^c^	211 (14.4%)	139 (12.7%)	72 (19.7%)	0.001
Type B (incomplete) ^c^	185 (12.7%)	99 (9.0%)	86 (23.5%)	<0.001
Type C (complete) ^c^	36 (2.5%)	17 (1.6%)	19 (5.2%)	<0.001
Femur fracture	269 (18.4%)	169 (15.4%)	100 (27.3%)	<0.001
**Hospital outcomes**				
ICU stay, days	4.0 (1.0, 11.0)	3.0 (1.0, 9.0)	5.5 (1.0, 16.0)	0.008
Hospital stay, days	21.0 (10.0, 38.0)	20.5 (11.0, 36.0)	22.0 (2.0, 51.5)	0.337
In-hospital mortality	239 (16.3%)	102 (9.3%)	137 (37.4%)	<0.001

Data are presented as median (Q1, Q3) for continuous variables and *n* (%) for categorical variables. Continuous variables were compared using the Wilcoxon rank-sum test and categorical variables using the chi-square test or Fisher’s exact test, as appropriate. Denominators vary for variables with missing data. ^a^ Restricted to patients who received any red blood cell transfusion within 24 h (MT, *n* = 366; non-MT, *n* = 421). The registry truncates this interval at 24 h. Eleven patients received plasma or platelets without red cells and are not included. ^b^ Measured on a complete blood count from a separate venous sample; lactate and ionized calcium were measured on the arterial blood gas analyzer in the trauma resuscitation room. ^c^ Pelvic fractures were classified as AO/OTA type A, B, or C from the Abbreviated Injury Scale code assigned by trauma surgeons during registry coding. AIS, Abbreviated Injury Scale; ED, emergency department; FAST, focused assessment with sonography for trauma; ICU, intensive care unit; MT, massive transfusion.

**Table 2:** Univariable logistic regression analysis for massive transfusion.

Characteristic	N Used	MT Events	OR (95% CI)	*p*-Value
**Demographics**				
Age (per 10 years)	1462	366	0.96 (0.90–1.03)	0.261
Male sex	1462	366	0.75 (0.57–0.98)	0.031
Penetrating injury	1462	366	6.43 (3.48–12.42)	<0.001
**Vital signs**				
Systolic blood pressure, mmHg	1392	317	0.97 (0.96–0.97)	<0.001
Diastolic blood pressure, mmHg	1391	316	0.95 (0.95–0.96)	<0.001
Heart rate, beats/min	1441	353	1.03 (1.02–1.03)	<0.001
Respiratory rate, breaths/min	1440	352	1.09 (1.06–1.11)	<0.001
Oxygen saturation, %	1438	350	0.93 (0.91–0.94)	<0.001
Body temperature, °C	1462	366	0.61 (0.52–0.71)	<0.001
Glasgow Coma Scale	1462	366	0.91 (0.88–0.93)	<0.001
**FAST findings**				
FAST: hemothorax	1462	366	6.61 (3.26–14.23)	<0.001
FAST: pneumothorax	1462	366	3.16 (2.17–4.60)	<0.001
FAST: abdomen	1462	366	5.79 (3.97–8.51)	<0.001
FAST: pelvis	1462	366	5.01 (2.09–12.74)	<0.001
Overall FAST positive	1462	366	4.88 (3.68–6.48)	<0.001
**Fractures**				
Pelvic fracture	1462	366	3.09 (2.41–3.96)	<0.001
Unstable pelvic fracture	1462	366	3.40 (2.52–4.58)	<0.001
Femur fracture	1462	366	2.06 (1.55–2.73)	<0.001
**Laboratory**				
pH (per 0.1 unit)	1434	366	0.49 (0.43–0.55)	<0.001
Base excess, mmol/L	1419	355	0.80 (0.77–0.82)	<0.001
Lactate, mmol/L	1434	366	1.43 (1.36–1.50)	<0.001
Hemoglobin, g/dL	1462	366	0.69 (0.65–0.73)	<0.001
Platelet count (per 10 × 10^3^/μL)	1462	366	0.97 (0.96–0.99)	0.001
Ionized calcium (per 0.1 mmol/L)	1431	366	0.22 (0.18–0.27)	<0.001

Odds ratios are presented with 95% confidence intervals. For continuous variables, age is expressed per 10-year increase, pH per 0.1-unit increase, platelet count per 10 × 10^3^/μL increase, and ionized calcium per 0.1 mmol/L increase. MT, massive transfusion; FAST, focused assessment with sonography for trauma; CI, confidence interval.

**Table 3:** Multivariable logistic regression analysis for massive transfusion.

Characteristic	Adjusted OR (95% CI)	*p*-Value
Age (per 10 years)	1.09 (0.98–1.21)	0.117
Male sex	1.47 (0.94–2.31)	0.096
Penetrating injury	5.16 (2.00–13.78)	<0.001
Systolic blood pressure, mmHg	0.98 (0.97–0.99)	<0.001
Heart rate, beats/min	1.01 (1.01–1.02)	<0.001
Respiratory rate, breaths/min	1.03 (1.00–1.07)	0.038
Glasgow Coma Scale	0.93 (0.89–0.97)	<0.001
Lactate, mmol/L	1.11 (1.03–1.20)	0.004
Hemoglobin, g/dL	0.79 (0.71–0.87)	<0.001
Ionized calcium (per 0.1 mmol/L)	0.37 (0.29–0.47)	<0.001
Overall FAST positive	2.33 (1.53–3.55)	<0.001
Pelvic fracture	1.99 (1.38–2.85)	<0.001

Adjusted odds ratios are presented with 95% confidence intervals. For continuous variables, age is expressed per 10-year increase and ionized calcium per 0.1 mmol/L increase. MT, massive transfusion; CI, confidence interval.

**Table 4:** ROC-derived cutoff values for continuous predictors.

Characteristic	AUC (95% CI)	Cutoff	Sensitivity	Specificity
Systolic blood pressure, mmHg	0.792 (0.762–0.822)	109.50	0.656	0.821
Heart rate, beats/min	0.693 (0.659–0.726)	104.50	0.575	0.744
Glasgow Coma Scale	0.621 (0.588–0.653)	13.50	0.555	0.641
Lactate, mmol/L	0.781 (0.754–0.809)	4.05	0.631	0.795
Hemoglobin, g/dL	0.696 (0.664–0.728)	12.25	0.514	0.790
Ionized calcium, mmol/L	0.787 (0.758–0.816)	1.02	0.571	0.880

Optimal cutoff values were determined using the Youden index. AUC, area under the receiver operating characteristic curve; CI, confidence interval.

**Table 5:** Development and comparison of candidate prediction models for massive transfusion.

Model	*n*	Events	AUC (95% CI)	Sensitivity	Specificity
Model A: full 9-item score	1356	317	0.864 (0.842–0.887)	0.707	0.865
Model B: 6-item score	1356	317	0.850 (0.826–0.874)	0.814	0.721
Model C: 5-item score	1356	317	0.845 (0.821–0.869)	0.808	0.723
Model D: 4-item score	1356	317	0.838 (0.813–0.862)	0.732	0.806
Model E: 3-item score	1364	317	0.802 (0.775–0.830)	0.852	0.609

Model A included penetrating injury, SBP ≤ 110 mmHg, HR ≥ 105 beats/min, GCS ≤ 13, hemoglobin ≤ 12.0 g/dL, lactate ≥ 4.0 mmol/L, ionized calcium ≤ 1.05 mmol/L, FAST positivity, and pelvic fracture. Model B included penetrating injury, SBP ≤ 110 mmHg, lactate ≥ 4.0 mmol/L, ionized calcium ≤ 1.05 mmol/L, FAST positivity, and pelvic fracture. Model C included SBP ≤ 110 mmHg, lactate ≥ 4.0 mmol/L, ionized calcium ≤ 1.05 mmol/L, FAST positivity, and pelvic fracture. Model D included SBP ≤ 110 mmHg, lactate ≥ 4.0 mmol/L, ionized calcium ≤ 1.05 mmol/L, and FAST positivity. Model E included SBP ≤ 110 mmHg, lactate ≥ 4.0 mmol/L, and FAST positivity. Confidence intervals for the AUC were obtained by the DeLong method. Bootstrap confidence intervals for the same models are given in Supplementary Table S2. AUC, area under the receiver operating characteristic curve; CI, confidence interval; FAST, focused assessment with sonography for trauma; GCS, Glasgow Coma Scale; HR, heart rate; MT, massive transfusion; SBP, systolic blood pressure.

**Table 6:** Comparison of the SMT, TASH, Modified TBSS, and ABC scores in the common complete-case cohort.

Score	*n*	Events	AUC (95% CI)	Cutoff	Sensitivity	Specificity	PPV	NPV	Accuracy
SMT score	1352	315	0.846 (0.822–0.870)	≥2	0.810	0.722	0.470	0.926	0.743
TASH score	1352	315	0.841 (0.815–0.868)	≥8	0.692	0.844	0.574	0.900	0.808
Modified TBSS ^a^	1352	315	0.821 (0.793–0.850)	≥14	0.663	0.838	0.554	0.891	0.797
ABC score	1352	315	0.783 (0.754–0.812)	≥2	0.381	0.968	0.784	0.837	0.831

All four prediction scores were evaluated in the same complete-case cohort (*n* = 1352), restricted to patients with data available for every variable required by all scores. Discrimination was assessed by the area under the receiver operating characteristic curve and compared using the paired DeLong test. Pairwise DeLong comparisons: SMT score vs. TASH score, *p* = 0.668; SMT score vs. Modified TBSS, *p* = 0.015; SMT score vs. ABC score, *p* < 0.001; TASH score vs. Modified TBSS, *p* = 0.058; TASH score vs. ABC score, *p* < 0.001; Modified TBSS vs. ABC score, *p* = 0.010. ^a^ The original TBSS requires systolic blood pressure measured after rapid infusion of 1000 mL of crystalloid, which cannot be reconstructed retrospectively. The Modified TBSS, which uses systolic blood pressure on arrival with the same scoring algorithm, was therefore calculated instead. The cutoff for the SMT score (≥2) was derived in this cohort. Cutoffs for the other scores were those originally reported: ≥2 for ABC, ≥8 for TASH, and ≥14 for the Modified TBSS. Confidence intervals for the AUC were obtained by the DeLong method, and AUCs were compared using the paired DeLong test. Bootstrap confidence intervals for the same cohort are given in Supplementary Table S4. ABC, Assessment of Blood Consumption; AUC, area under the receiver operating characteristic curve; CI, confidence interval; NPV, negative predictive value; PPV, positive predictive value; SMT, Simplified Massive Transfusion; TASH, Trauma-Associated Severe Hemorrhage; TBSS, Traumatic Bleeding Severity Score.

**Table 7:** Cutoff-specific performance of the SMT score.

Cutoff	TP	TN	FP	FN	Sensitivity	Specificity	PPV	NPV	Accuracy
≥1	305	422	617	12	0.962	0.406	0.331	0.972	0.536
≥2	256	751	288	61	0.808	0.723	0.471	0.925	0.743
≥3	196	937	102	121	0.618	0.902	0.658	0.886	0.836
≥4	94	1019	20	223	0.297	0.981	0.825	0.820	0.821
≥5	26	1033	6	291	0.082	0.994	0.812	0.780	0.781

Calculated in the complete-case cohort for the five SMT components (*n* = 1356; 317 events). Table 6 reports the same score in the common complete-case cohort (*n* = 1352). FN, false negative; FP, false positive; NPV, negative predictive value; PPV, positive predictive value; SMT, Simplified Massive Transfusion; TN, true negative; TP, true positive.

## Data Availability

The data presented in this study are available on request from the corresponding author. The data are not publicly available due to institutional and patient privacy restrictions.

## Supplementary Materials

The following supporting information can be downloaded at: https://www.mdpi.com/article/10.3390/jcm15166344/s1, Table S1: Missing data for physiological and laboratory variables, overall and by outcome group; Table S2: Composition and discrimination of the five candidate models; Table S3: Internal validation of the SMT score by bootstrapping with 1000 replicates; Table S4: Sensitivity analysis using alternative definitions of massive transfusion.
