# Supplementary material for: Development and Validation of a Simplified Five-Variable Score for Predicting Massive Transfusion in Blunt-Trauma-Predominant Patients
Source: J Clin Med. 2026 Aug 17;15(16):6344. doi: 10.3390/jcm15166344 (PMC13513371; doi:10.3390/jcm15166344)
Supplement: Supplementary file 1 [file jcm-15-06344-s001.zip › jcm-4471320-supplementary.pdf]

Table S1. Missing data for physiological and laboratory variables, overall and by outcome group.

Variable	Overall, n (%)	Non-MT (n = 1,096)	MT (n = 366)	p-value
Systolic blood pressure	70 (4.8)	21 (1.9)	49 (13.4)	<0.001
Diastolic blood pressure	71 (4.9)	21 (1.9)	50 (13.7)	<0.001
Heart rate	21 (1.4)	8 (0.7)	13 (3.6)	<0.001
Respiratory rate	22 (1.5)	8 (0.7)	14 (3.8)	<0.001
Oxygen saturation	24 (1.6)	8 (0.7)	16 (4.4)	<0.001
Body temperature	0 (0.0)	0 (0.0)	0 (0.0)	–
Glasgow Coma Scale	0 (0.0)	0 (0.0)	0 (0.0)	–
pH	28 (1.9)	28 (2.6)	0 (0.0)	<0.001
Base excess	43 (2.9)	32 (2.9)	11 (3.0)	1.000
Lactate	28 (1.9)	28 (2.6)	0 (0.0)	<0.001
Ionized calcium	31 (2.1)	31 (2.8)	0 (0.0)	<0.001
Hemoglobin	0 (0.0)	0 (0.0)	0 (0.0)	–
Platelet count	0 (0.0)	0 (0.0)	0 (0.0)	–

Values are the number (%) of patients with the variable missing. Groups were compared using the chi-square test or Fisher's exact test, as appropriate.

Lactate and ionized calcium were not missing in any patient who received massive transfusion. Systolic blood pressure showed the opposite pattern, being missing more often in the MT group.

Abbreviations: MT, massive transfusion.

Table S2. Composition and discrimination of the five candidate models.

Model	Variables	Components	N	AUC (95% CI)
Model A	9	Penetrating injury, SBP ≤110, HR ≥105, GCS ≤13, Hb ≤12.0, lactate ≥4.0, iCa ≤1.05, FAST+, pelvic fracture	1,356	0.864 (0.841–0.886)
Model B	6	Penetrating injury, SBP ≤110, lactate ≥4.0, iCa ≤1.05, FAST+, pelvic fracture	1,356	0.850 (0.825–0.874)
Model C (SMT score)	5	SBP ≤110, lactate ≥4.0, iCa ≤1.05, FAST+, pelvic fracture	1,356	0.845 (0.820–0.869)
Model D	4	SBP ≤110, lactate ≥4.0, iCa ≤1.05, FAST+	1,356	0.838 (0.812–0.862)
Model E	3	SBP ≤110, lactate ≥4.0, FAST+	1,364	0.802 (0.776–0.829)

Each component contributes one point. N varies because each model was evaluated using complete-case data for the variables it requires. Confidence intervals were obtained by bootstrapping with 2,000 replicates and therefore differ slightly from the DeLong intervals reported in Table 5.

Model C was selected as the final model on the basis of discrimination and the number of variables required at the bedside. Penetrating injury was excluded by design so that the score carries no assumption about mechanism of injury.

Abbreviations: AUC, area under the receiver operating characteristic curve; CI, confidence interval; FAST, focused assessment with sonography for trauma; GCS, Glasgow Coma Scale; Hb, hemoglobin; HR, heart rate; iCa, ionized calcium; SBP, systolic blood pressure; SMT, Simplified Massive Transfusion.

Table S3. Internal validation of the SMT score by bootstrapping with 1,000 replicates.

Approach	Apparent AUC	Optimism	Optimism-corrected AUC
Logistic model, five binary items	0.853	0.003	0.851
Unweighted integer score (0–5)	0.845	<0.001	0.845
Full pipeline, cutoffs re-derived per replicate	0.856	0.004	0.852

Calibration measure	Value
Calibration slope	0.986
Calibration-in-the-large	0.000
Brier score	0.119

Optimism was estimated by Harrell's method. In the third approach, the Youden-based cutoffs for continuous variables were re-derived within each bootstrap replicate, so that the full model-development pipeline rather than the fixed score was validated.

Analyses used the complete-case cohort for the five SMT components (n = 1,356; 317 events).

Abbreviations: AUC, area under the receiver operating characteristic curve; SMT, Simplified Massive Transfusion.

Table S4. Sensitivity analysis using alternative definitions of massive transfusion.

Definition of MT	Events	SMT score	TASH score	Modified TBSS	ABC score
Primary (≥4 U/4 h or ≥10 U/24 h)	315	0.846 (0.821–0.870)	0.841 (0.814–0.866)	0.821 (0.792–0.849)	0.783 (0.755–0.814)
≥10 U within 24 h only	120	0.830 (0.793–0.865)	0.821 (0.778–0.859)	0.792 (0.744–0.838)	0.789 (0.745–0.832)
≥4 U within 4 h only	307	0.849 (0.824–0.873)	0.846 (0.821–0.871)	0.826 (0.798–0.853)	0.784 (0.756–0.814)

Values are the AUC (95% CI) in the common complete-case cohort (n = 1,352). Confidence intervals were obtained by bootstrapping with 2,000 replicates and therefore differ slightly from the DeLong intervals reported in Table 6.

Discrimination was slightly lower under the more restrictive 24-hour definition, as expected with fewer events, but the relative ordering of the scores was preserved under all three definitions.

Abbreviations: ABC, Assessment of Blood Consumption; AUC, area under the receiver operating characteristic curve; CI, confidence interval; MT, massive transfusion; SMT, Simplified Massive Transfusion; TASH, Trauma-Associated Severe Hemorrhage; TBSS, Traumatic Bleeding Severity Score.
